# A comprehensive analysis of time investment in skid trail planning for forest access

**DOI:** 10.1371/journal.pone.0317963

**Published:** 2025-02-04

**Authors:** Marc Werder, Leo Gallus Bont, Janine Schweier, Oliver Thees

**Affiliations:** Swiss Federal Institute for Forest, Snow and Landscape Research (WSL), Birmensdorf, Switzerland; Chang’an University, CHINA

## Abstract

Properly planned skid trails form an important basis for sustainable timber production. They affect cost-effectiveness and the environmental impact of the harvesting process to a large extent. Here, we conducted an economic analysis to understand the skid trail planning process and to generate an initial model to estimate the time and costs involved. We investigated in detail how the planning process of skid trails is carried out in practice, what time is required for the planning work, and what factors influence its performance. Through an online survey conducted in 2022, we asked practitioners in Germany and Switzerland about their time and effort required for the planning process and the determining factors, such as the planning method and the terrain and stand conditions. Based on this survey, we calculated statistical indicators of time consumption, considered possible rationalization options, and developed an initial estimation model. The effort required to identify and evaluate skid trails planned for distances of 20 to 40 m amounts to around 3 to 4 hours of productive working time per hectare, with deviations expected depending on the specific situation in the forest. The costs corresponding to this investment amount to less than one euro per cubic meter of harvested timber, depending primarily on the extent of wood use. Our in-depth insight into the planning process enables its economic evaluation and the development of improvements.

## 1. Introduction

Skid trails are a prerequisite for the sustainable management of forests in accessible terrain in order to concentrate the machine driving necessary for interventions in these areas. A suitable network of skid trails enables cost-efficient and environmentally friendly timber harvesting, in particular the minimization of soil damage. The latter aspect has gained in importance, as the mechanization of timber harvesting has led to the use of heavier machinery, increasing the risk of soil damage, such as compaction through machines during operation. When planning skid trails, it is therefore becoming increasingly important to consider both ecological and economic aspects. To complicate matters further, the progressing development of harvesting technology and increasing ecological requirements lead to the fact that, besides the installation of new skid trails, existing ones must be integrated into the new network–including those that are damaged or need to be restored. These planning projects therefore involve considerable complexity and quality requirements, demanding a corresponding amount of work. Although it is possible to find optimal solutions with the help of modern technologies, heuristic approaches, which are usually implemented with the help of geographic information systems (GIS), still seem to dominate in practice.

While the quality of the planning and particularly its effects, e.g. on timber harvesting performance and environmental aspects, have been studied extensively, the process of sophisticated planning of skid trails as it occurs in practice and the associated costs have hardly been investigated scientifically.

For decades, research has repeatedly focused on the economic and ecological aspects of skid trail networks. The focus has been on the effects of skid trails in terms of the cost efficiency of timber harvesting and the risks to soil protection. However, economic considerations or the time and costs of this demanding planning process itself have received little attention. For example, Garland [1] pushed the topic of skid trails in the United States already in the early 1980s. In the following decades, there was extensive related research. An overview of the impact of heavy traffic on forest soils was provided by Cambi et al. [2], and Labelle et al. [3] published a review on the strategies to mitigate the effects. Since the beginning of the 2010s, more studies have been conducted on the optimal and the automatically designed layout of skid trail networks [4–11]. Regardless, heuristic approaches continue to dominate in practice and, accordingly, practical guidelines or practice codes have been developed in the respective national languages [12–14]. To summarize, as the time and the associated costs required for planning skid trails received little to no attention in academic work, hardly any quantitative information is available nationally or internationally to estimate the time required for this process [14–17].

In the European understanding, skid trails are unstocked access lines of 3 to 4 m width without ground movement or fixing/pavement, built in forest stands for logging machinery. They are carefully planned as a permanent system of–if possible–parallel lines at distances of 20 to 40 m apart, and their planning considers technical, economic, environmental and social aspects. In this context, permanent is defined as an expected period of use of about 50 years or more. It should be noted that, unlike forest roads, skid trails are mostly considered part of the productive forest area.

Against this background, the aim of this empirical study was to determine the time required for planning skid trials and to develop an initial model for estimating the effort involved. For this purpose, we assessed the economic aspects of the planning process of skid trails through an online survey conducted in Switzerland and Germany, targeting the forestry managers responsible for this process. The analysis did not include an evaluation of the quality or the effectiveness of the planning. Based on the survey, we aimed to: (i) analyze which methods are used in practice for planning skid trails, (ii) identify the key factors determining the time and cost requirements for planning, and (iii) develop a prototype model that enables estimations of the time and associated costs for future planning activities.

## 2. Methods

### 2.1 Online survey and data collection

An online survey was conducted using a standardized questionnaire in forestry practice to gain insights into the current situation of skid trail planning. This was done to better understand the planning process and to set the initial basis for estimating the time and cost investments or requirements of skid trail planning. The survey included questions about the time required for planning activities and the potential factors affecting the performance, such as the planning methods employed, the design of the skid trail network to be planned, the terrain and stand conditions, the planner’s experience, and the use of an additional labor force.

The questionnaire was created with the professional online tool “LimeSurvey” [18] and pretested by three selected skid trail planning experts from Germany and Switzerland for qualitative verification before distribution. After their feedback was incorporated, the survey was sent out in June 2022 and was online for six months. The target group was selected forestry managers who are responsible for skid trail planning and have extensive experience. In Switzerland and Germany, well-known specialists were asked to take part in the survey and to forward the questionnaire to other experts (snowball approach). The survey was conducted in the Swiss cantons that, according to the National Forest Inventory (NFI) [19], have a high proportion of trafficable forest terrain in terms of surface area, i.e. land that can be accessed through skid trails (mainly the midlands, e.g. the cantons of Aargau, Bern, Lucerne, Schaffhausen, Zug and Zurich). In Germany, a country with a large proportion of trafficable forest terrain, survey participants were primarily recruited with the support of the German Centre for Forest Work and Technology (KWF) in Gross-Umstadt. The network of this specialist organization made it possible to identify expert practitioners in the state forest administrations of Baden-Württemberg, Bavaria, Lower Saxony, Rhineland-Palatinate and Thuringia for participation in the survey.

Approximately 200 questionnaires were sent out to forest managers by the Swiss Federal Institute for Forest, Snow and Landscape Research (WSL) and the aforementioned partners. Of these, 44 were fully completed, which came very close to the goal of obtaining approximately 50 analyzable responses. The questionnaire can be found in S1 File.

### 2.2 Time consumption

The time requirement for skid trail planning forms an essential basis for time management within a forest enterprise and for the assessment of costs. It thus constituted the central variable of the survey. Survey participants estimated the time required for their planning work in terms of productive system hours (PSH_15_), i.e. the working hours per hectare including all interruptions <15 minutes. This was done depending on the working conditions for each of the five work steps of the planning process (see section 2.3.1: Planning methods, Table 1). In cases where additional labor was used for the planning process (e.g. assistants for field work), the corresponding time requirement was considered based on the working conditions for each of the five work steps.

**Table 1:** Overview of the five defined steps involved in planning skid trails.

Planning step	Planning category	Description
1	Preparation in the office	**Defining of the perimeter to be planned** under consideration of the higher-level planning entity and **identification of the main aspects**, particularly all existing skid trails, with the documents available in the office (e.g. topographic maps, satellite images, orthophotos, lidar images).
2	Preparation on site	**Site inspection** (as a rule), e.g. to record missing trails or to check trafficability at critical points.
3	Planning	**Planning and mapping of the new skid trail system** according to a concept of best practice (e.g. skid trail direction, spacing of the trails), where as many existing skid trails as possible should be adopted regarding soil protection.
4	Marking	**Verification and marking of the newly planned skid trails** in the forest (e.g. adjusted layout of the skid trails caused by obstacles, microtopography, wet spots), which is done either by hand (paper map, sight pole and compass), with a mobile GPS, or in combination (Fig *1*).
5	Documentation	**Documentation of the recorded skid trail networks**, carried out in the office.

### 2.3 Factors influencing productivity

#### 2.3.1 Planning methods

**Fig 1:**
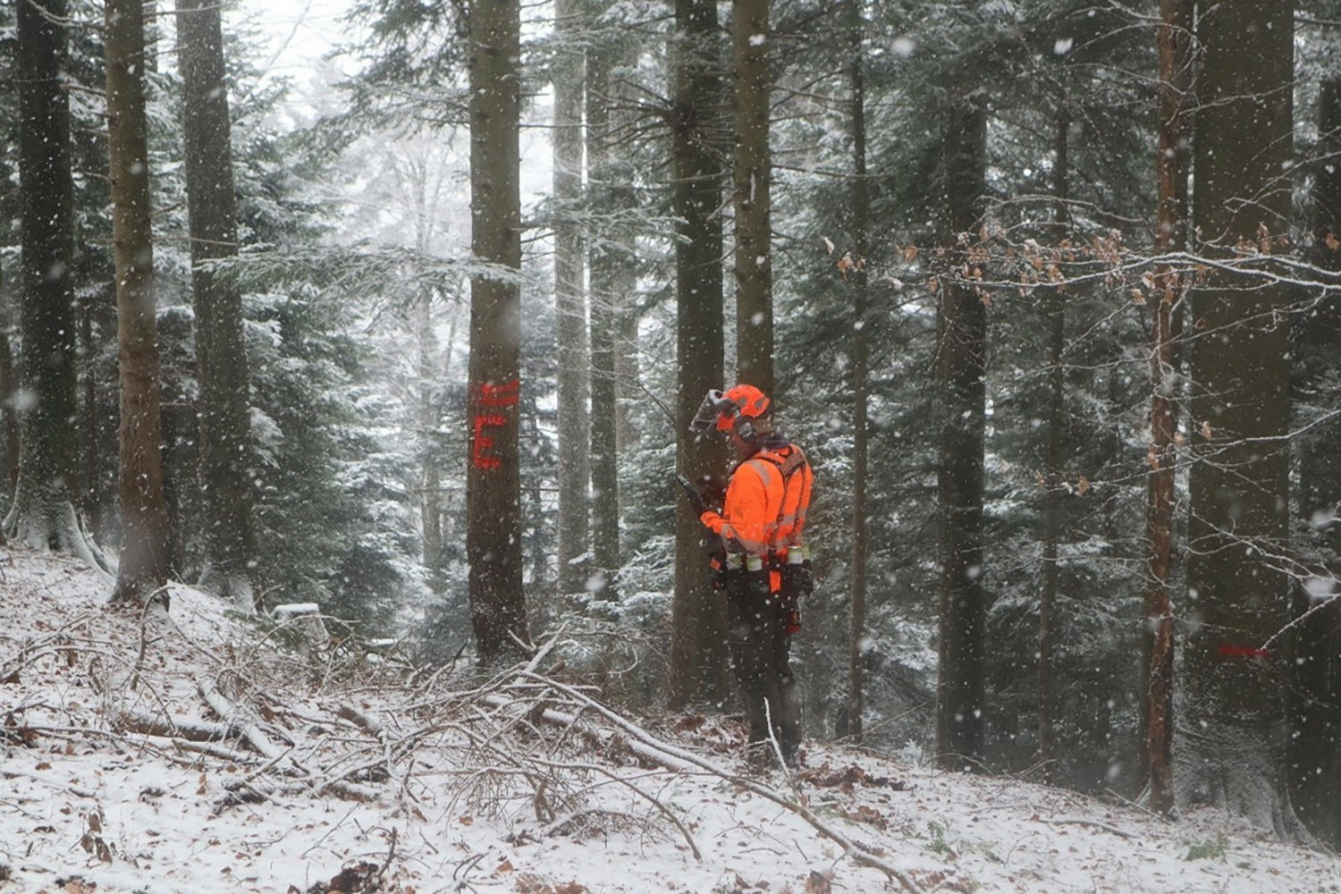
Forester marking the end of a skid trail with a mobile geographic positioning system (planning step 4). Photo: M. Werder, WSL. Published under a CC BY license.

There are numerous ways in which the planning of a skid trail network can be done. These range from traditional approaches, characterized by manual labor, to modern methods involving information technologies (e.g. a geographic positioning system (GPS), cf. Fig 1). Accordingly, a variety of methods are used in practice. So far, there are no commonly used and clearly defined terms for the methods used. Nevertheless, a basic and technology-neutral survey procedure could be delineated through the five steps described in Table 1, which were defined with the help of experts prior to the investigation. According to this structure, survey participants were asked to describe the method they usually apply.

#### 2.3.2 Terrain and stand conditions

The survey participants were asked to estimate the time requirement for each step of the planning process under various terrain and stand conditions (Table 1). For this purpose, the following three levels of condition difficulty were distinguished based on the experts’ recommendations prior to the investigation:

- Easy conditions: flat terrain, no foliage, clear, very good visibility, no or few obstacles from vegetation and terrain features such as ditches and rocks.
- Moderate conditions: medium slope and ground roughness, spring or fall timing with little foliage, young forest passages, limited visibility.
- Difficult conditions: pronounced slope, leafy condition, large proportion of young forest or pole wood, large proportion of coniferous wood, considerably restricted visibility, high ground roughness, wet spots, many old skid trails to consider.

#### 2.3.3 Distances between skid trails

The distances between skid trails influence the ecological and economic impact of timber harvesting considerably, forming a well-recognized trade-off relationship. In this conflict, the main aim is to find an optimal trail spacing that ensures both soil protection and feasible harvesting costs. Depending on the sensitivity of the soil to compaction and the conditions of the stand and its intended use, various skid trail distances are planned and implemented in forest districts, with a trend toward larger distances and with corresponding effects on the planning effort. For instance, Frutig et al. [7] suggested that an optimal distance between skid trails in Switzerland ranges from 30 to 50 m. Regarding planning efforts, the shorter the distance between skid trails, the greater the total length of the trails and the corresponding planning effort per unit area. Due to this relationship, the surveyed managers were asked about the frequency distribution of skid trail distances in their planning, and the weighted mean of the planned skid trail distances was determined from this information for further use in the regression analysis.

#### 2.3.4 Field work assistants

The planning of skid trails is a task typically conducted by the forest district managers, (especially in Germany) and usually without the assistance of additional personnel. Mainly under challenging terrain and stand conditions, it may be necessary to involve a second person in the field work during step 4 –verification and marking of the newly planned skid trails (Table 1). This necessity arises from the estimate by forest district management that the task is not feasible alone or that it can be carried out more efficiently and cost-effectively with support.

Therefore, the survey included a question about whether an assistant is employed and, if yes, under what conditions, during which steps of the planning process, and for how long. Survey participants were asked to report the time in personnel hours (PPH) or system hours (PSH) of productive working time, including interruptions of less than 15 minutes (PPH_15_ or PSH_15_).

#### 2.3.5 Experience of the planners

It can be assumed that experienced planners require less time for the task, thus planning more efficiently than their less experienced colleagues. Hence, survey participants were asked about the number of years of experience they have in skid trail planning. As experience depends not only on the number of years but also on the annual workload (measured in hectares of forest) for this task, this metric was also considered. From both pieces of information, a variable for the regression analysis was constructed in the form of the product of years of experience and hectares per year.

### 2.4 Statistical analysis

After the survey was completed, the data were collated and analyzed descriptively.

#### 2.4.1 Regression models

Two regression model categories were applied to derive models for predicting productivity: common ordinary least squares (OLS) regression models and robust (ROB) regression models. The two kinds of models were applied because an initial inspection of the data showed many possible outliers. OLS regression provides perfect estimators when the data are exactly normally distributed. Real data are never exactly normally distributed, however, and even a single outlier can mean that OLS regressions become ineffective for parameter estimations. Therefore, ROB regression was incorporated because this method can confidently identify and weight outliers.

The OLS regression model formulation is defined in Eq 1:

$$Y(x)=\beta_{0}+\beta_{1}Z_{1}+\beta_{2}Z_{2}+..+\beta_{p}Z_{p}+\varepsilon (Z)$$ (1)

where *Y*(*x*) is the response variable, *β*_0_..*β*_*p*_ are the regression coefficients, *Z*_1_..*Z*_*p*_ denote the predictor variables, and *p* is the number of predictor variables. The error term *ε*(*Z*) is taken to be independent and identically distributed, *E*(*ε*(*Z*)) = 0 and *Var*(*ε*(*Z*)) = *σ*^2^. All models are displayed using the following formatting: *Y*~*Z*_1_+*Z*_2_+..+*Z*_*p*_ (formulation used in R software).

The ROB regression analysis was done with the ‘lmrob’ function from the *robustbase* package [20] in R. The default settings were used: a bi-square redescending score function, which returns a highly robust and highly efficient estimator (with 50% breakdown point and 95% asymptotic efficiency for normal errors). The function ‘lmrob’ computes an MM-type regression estimator, as described by Yohai [21] and by Koller and Stahel [22]. Leave-one-out cross-validation (LOOCV) was used for model assessment and evaluation.

#### 2.4.2 Model selection

The initial model that was tested was the complete model, containing all predictors that are listed in Table 2 (see also S1 Table) but without interaction terms (step 1). As *PMS* (planning method simplified) is a simplified version of *PMF* (planning method full), these two variables were never incorporated in the same model. To find a satisfactory balance between the goodness of fit and the simplicity of the model (to avoid overfitting), a further model formulation was evaluated by performing variable selection based on the Akaike information criterion (AIC, [23] step 2). Next, a model diagnosis was done (step 3). This was an essential step to take before any findings from the summary output, confidence intervals or predictions could be interpreted. Model diagnosis involved checking the error assumptions using residual analysis and was done by visually analyzing a normal plot, a Tukey-Anscombe plot, a scale-location plot, a leverage plot, and a plot of each potential predictor versus the residuals.

**Table 2:** Overview of the response and predictor variables included in the models.

Response (= R) / predictor (= P) variable	Label
Forester working time (R)	*Time_F*
Total working time (R)	*Time_tot*
Planning method (full) (P)	*PMF*
Planning method simplified (P)	*PMS*
Terrain and stand conditions (P)	*Cond*
Skid trail spacing (P)	*STS*
Field work assistant (P)	*FWA*
Total experience as total planned area (P)	*EXP_area*
Current practice (P)	*Current_Pract*
Total experience as total years of experience (P)	*EXP_years*

If the error assumptions were not fulfilled, two options were pursued. Option 1 (step 4) was to add meaningful interaction terms. For example, a potentially meaningful interaction term was between the incorporation of a field work assistant (FWA) and the planning method, such as *PMS* or *PMF*. Option 2 (step 5) was to check whether a predictor or the response variable required a so-called ‘first-aid’ transformation (log, square-root or arcsine). In this case, the model fitting started anew, i.e. steps 1 to 3 were performed again.

#### 2.4.3 Model assessment

At the deadline (31.12.2022), the questionnaire had been completed by a total of 55 participants. Of these, 30 were from Switzerland and 25 from Germany. However, 11 questionnaires were not filled out completely, resulting in a final count of 44 for the statistical analysis apart from the regression analysis. For modeling purposes, the set of 44 participants was divided into 2 groups according to the methods applied. Three questionnaires could not be assigned and were excluded, leaving 41 cases ultimately used for regression analysis (see section 3.2.1: Planning methods and section 3.3: Regression models).

## 3. Results

### 3.1 Consumption and distribution of time

The productive system hours (PSH_15_) required to complete a skid trail planning process were, on average, 3.62±2.53 PSH_15_ ha^-1^ (Table 3). Planning step 4 –verification and marking of the newly planned skid trails in the forest–was the most time-consuming task (60.5%). The high magnitude of the calculated standard deviation for the complete planning process, as well as within the planning steps, indicates large variation in the data. Apparently, the estimated time requirements were skewed because some participants indicated very high time requirements. The median (2.9 PSH_15_ ha^-1^) is therefore a more stable, realistic value to describe the distribution (Table 3).

**Table 3:** Time required for planning tasks (n = 44). PSH_15_ = productive system hours per hectare, including all interruptions <15 minutes.

Planning step	Planning category	Mean value ± st. dev. (PSH_15_)	Proportion (% PSH_15_)	Median (PSH_15_)
1&2	Preparation	0.65±0.06	17.96	0.50
3	Planning	0.42±0.03	11.60	0.25
4	Marking	2.19±0.14	60.50	2.00
5	Documentation	0.36±0.03	9.94	0.25
	Total time	3.62±0.22	100.00	3.00

### 3.2 Description of the analyzed factors

#### 3.2.1 Planning methods

Ten distinct but partially related planning methods were derived from the participants’ answers, ranging from a completely traditional approach involving paper maps, ranging rods and compasses to a fully digitized method using only GIS and GPS technologies. Two planning methods occurred frequently and emerged as particularly noteworthy (Table 4).

**Table 4:** The two planning methods (3 and 5) described most frequently by the participants (used as the basis for the groups formed for the regression analysis) and average values of the estimated time requirements. PSH_15_ = productive system hours per hectare including all interruptions <15 minutes.

Planning step	Planning category	Planning methods	
		Method 3 (n = 21)	Method 5 (n = 11)
		(basis of group 1 “partly digitized")	(basis of group 2 “fully digitized")
1	Preparation in the office	Using topographic maps, satellite images, orthophotos and lidar images	
2	Preparation on site	GPS recording of missing points in the stand	
3	Designing	Planning and mapping the new skid trail system in the office using GIS	
4	Marking/Verification	Using a combination of paper maps, ranging rods, compasses and mobile GPS	Solely performed with mobile GPS
5	Documentation	Storing the fully planned skid trail entity	
**Total time required** PSH_15_ ha-1		**Group 1 “partly digitized”** (n = 27)	**Group 2 “fully digitized”** (n = 15)
Mean (standard deviation)		4.03 (±2.63)	2.75 (±2.03)
Median		3.40	2.25

As shown in Table 4, the only difference between the two groups in the planning process occurred in step 4. Whereas group 1 applies a partly digitized planning method while working in the stand, with the aid of “traditional” materials such as paper maps, site poles and compasses, the method of group 2 is fully digitized, solely involving mobile GPS. For the preparatory work and for the final documentation in the office (steps 1 and 5), only minor differences occurred.

Other combinations of planning methods were observed. Due to their resemblance to the two methods above, these combinations were grouped accordingly. Three participants carried out their planning entirely without using digital tools, and therefore their methods could not be categorized. To include the planning methods as a variable in the regression model, the responses from these three participants were removed, resulting in 27 participants in group 1 and 14 in group 2.

There was a significant difference between the time requirements for a partially (group 1) and a fully digitized planning process (group 2) (Mann-Whitney U-test; *P* = 0.003185). The time required for group 1 was around one hour (PSH_15_) longer than that for group 2. This difference can be seen both in the mean values and in the medians. For both methods and groups, the medians of the time required are around 30 to 45 minutes less than the mean values. Since the difference in the planning strategies between the two groups is in step 4, this step was analyzed separately statistically. The result was an average effort of 2.40±1.70 PSH_15_ ha^-1^ for group 1 compared with 1.81±1.36 PSH_15_ ha^-1^ for group 2. Thus, a fully digitized planning method appears to be approximately 0.6 PSH_15_ ha^-1^ quicker in verifying and marking the skid trails than a method with additional analog tools. The respective medians were 2.0 and 1.0 PSH_15_ ha^-1^.

#### 3.2.2 Terrain and stand conditions

When the time required for the entire planning process was examined for all the available data, across all terrain and stand conditions (easy, moderate, difficult), significant differences in the time consumption per hectare were found (Table 5).

**Table 5:** Total time required for the planning process under different terrain and stand conditions (n = 44). PSH_15_ = productive system hours per hectare including all interruptions <15 minutes.

	Time required for planning (PSH_15_ ha^-1^)						
Conditions	Min.	1st Qu.	Median	Mean	3rd. Qu.	Max.	p-value (t-test)
**Easy**	0.30	1.30	2.40	2.61±1.72	2.90	8.00	0.00
**Moderate**	0.49	2.18	3.25	3.47±2.07	4.63	10.00	0.00
**Difficult**	0.70	2.50	4.20	4.80±3.16	6.00	15.00	0.00

Thus, the planning process was approximately 2 hours ha^-1^ quicker under easy conditions than under difficult ones. The difference between moderate and difficult conditions was larger than that between easy and moderate, for both mean and median values (Table 5).

#### 3.2.3 Field work assistants

The use of a field work assistant (FWA) was concentrated almost exclusively in step 4 of the planning process (Table 1), i.e. in 36% of the cases (n = 16; 13 from Switzerland, 3 from Germany). Of these 16 cases, all survey participants utilized a FWA in difficult conditions, 15 in moderate conditions, and 12 in easy conditions. The time savings were not significantly different for the managers or for the respective system hours (PSH_15_): throughout the whole planning process, the average time requirement was 3.25±2.1 (median 2.60) PSH_15_ ha^-1^ with the support of a FWA and 3.84±2.75 (median 3.2) PSH_15_ ha^-1^ without one. The time savings amounted to an average of 18% or, in terms of medians, 23%; for both statistical measures, the absolute saved system time was 0.6 PSH_15_ ha^-1^.

When step 4 (marking and verification) was considered separately, the average time requirement was 2.3±1.54 PSH_15_ ha^-1^ without a FWA and 2.0±1.68 PSH_15_ ha^-1^ with a FWA, representing a system time savings of 15%. The median time requirement was 2.0 PSH_15_ ha^-1^ without a FWA and 1.5 PSH_15_ ha^-1^with a FWA, representing a system time savings of 33%.

Assuming the FWA spends the same amount of time on site in step 4 as the planning manager, the FWA would be on site for 2.00 (median 1.50) PSH_15_ ha^-1^ on average (62% and 58% of PSH_15_, respectively). This results in 5.25 (median 4.10) personnel hours (PPH_15_) ha^-1^ in total, which corresponds to approximately 1.41 (or 0.90) hours (PPH_15_) ha^-1^ more compared with planning without a FWA (S4 Table).

#### 3.2.4 Level of experience

**Fig 2:**
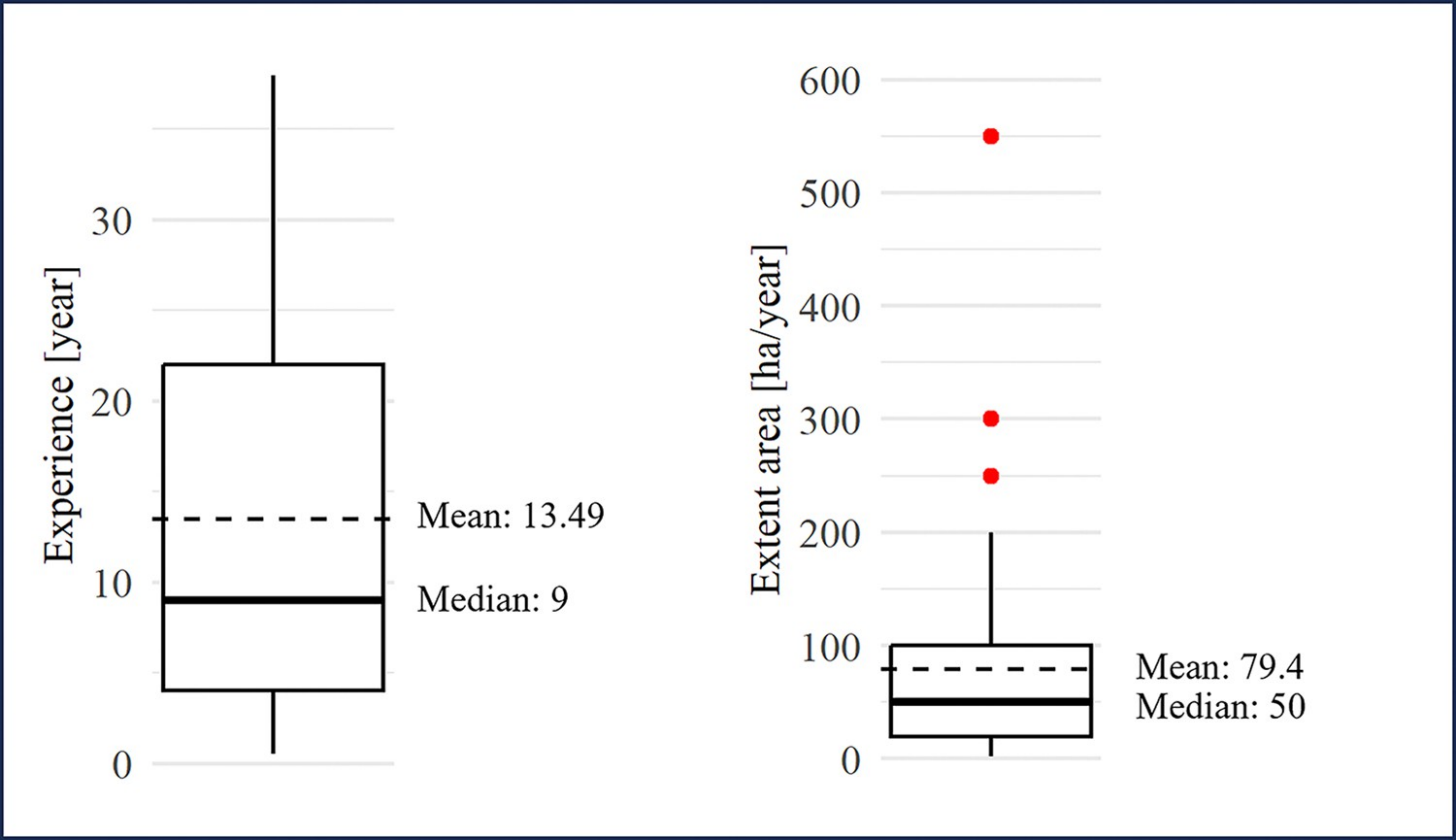
Experience (left) and annual work volume (extent area, right) of the planners.

Most of the survey participants had many years of experience (Fig 2), with values ranging from 0.5 to 38 years. Three-quarters of the participant’s annual work volume summed to less than 100 ha year^-1^; however, some participants reported very high values, between 200 and 600 ha year^-1^.

### 3.3 Regression models

All models were generated with a log-transformed response variable (*Time_F*), and most of the numeric predictors were log- or square-root (sqrt) transformed: sqrt (*EXP_years*, *years of experience*), log (*Current_Pract*, *annual work volume*), and sqrt (*EXP_area*, *total work volume*). Only *STS (skid trail distance spacing)* was not transformed in all cases: models with untransformed response and predictor variables showed significant problems in the model diagnostics (residual plots). All variables are defined in Table 2.

The model categories were created according to S1 Table, which included all combinations of the simplified (*PMS*) or full (*PMF*) planning method variable, without interaction terms, and with two simple and one full interaction term (S2 Table). Additionally, one expert-based model was created (Table 6, model 1a) to facilitate the usability.

**Table 6:** Model overview with predictors and key properties. The models are written in the following way in R (example given for model 1a): log(Time_F) ~ Cond + log(STS) + PMS + log(Current_Pract) + FWA + Current_Pract:FWA. The following significance codes apply: ‘***’ P = 0–0.001;‘**’ P = 0.001–0.01;‘*’ P = 0.01–0.05; ‘.’ P = 0.05–0.1; ‘()’ P = 0.1–1.

	Models	Predictors										Interaction terms				Model performance	
		[log = log transformed; sqrt = square root;— = not considered]															
		*Cond (Verh)*	*STS (RGA)*	*log(STS) (RGA)*	*PMF*	*PMS*	*GIS*	*sqrt(EXP_years) (Erfahrung)*	*sqrt(EXP_area) (Umfang_total)*	*log(Current_Pract) (Umfang)*	*FWA (Hilfskraft)*	*PM*: *FWA*	*PM*: *EXP_area*	*FWA*: *EXP_area*	*STS*: *EXP_years*	*adj*.*r*.*squared*	*LOOCV_RMSE_PERC*
1	*PMSwo*	***	-	-	-	***	-	***	***	***	.	-	-	-	-	0.33	61.37
**1a**	***PMSwo_Exp***	***	**-**	**	**-**	***	**-**	**-**	**-**	**	**()**	**	**-**	**-**	**-**	**0.30**	**62.81**
2	*PMSs1*	***	*	-	-	***	()	*	**	***	()	*	-	-	-	0.36	60.41
3	*PMSs2*	***	()	-	-	***	()	*	()	**	()	*	-	()	-	0.40	60.91
4	*PMSfu*	***	()	-	-	***	-	-	-	***	()	*	***	***	**	0.42	57.09
5	*PMFwo*	***	()	-	***	-	-	**	***	***	*	-	-	-	-	0.38	56.69
6	*PMFs1*	***	**	-	***	-	-	*	**	***	***	***	-	-	-	0.44	57.37
7	*PMFs2*	***	-	-	***	-	-	***	-	***	**	***	***	***	-	0.50	57.41
8	*PMFfu*	***	()	-	***	-	-	-	-	***	**	***	**	***	**	0.51	54.19

Table 6 shows the resulting models. The model performance, expressed as the adjusted r-squared value, varied between 0.30 and 0.51, leave-one-out cross-validation (LOOCV) resulted in scores between 54.2% and 62.8%.

In all the models, the predictors of terrain and stand conditions (*Cond*) and planning methods (partly and fully digitized; *PM*) were identified as significant.

The models with the full (*PMF*) planning method variable (models 5–8) performed better than those with the simplified variable (*PMS*). However, for an application, choosing between 10 different options (*PMF* with 10 levels) would overwhelm the users. Using the simplified variable *PMS* is more user friendly, at the price of only a slightly worse model performance. Further, models 3 and 4 (Table 6) include two and four interaction terms, respectively, and would therefore be difficult to interpret by the users. In the end, models 1 and 2 remained as potential models for predictive use. At this point, we wondered why the variable *STS* (skid trail spacing) disappeared and correlated strongly with *EXP_years*. From our point of view (and what we have learned from discussions with experienced planners), it makes sense to include *STS*. In the end, the expert-based model 1a (Table 6) showed the lowest model performance (slightly lower than the other models), but it had the advantage of being easily understandable and involving predictors that make also sense from an expert’s point of view. Also, the model diagnostic plot (S1 Fig) looks quite good.

In the end, we decided to use model 1a (Table 6) for prediction purposes. To get model predictor estimates that are robust toward potential outliers, we used the ROB model (see S3 Table for model summary output), which had only slight adaptations (of coefficients) compared with the non-robust variant. The model can be interpreted as follows:

The time required for skid trail planning increases with increasing condition difficulty, with decreasing skid trail spacing, with a smaller annual work volume, and when a partly digitized planning method is used.

The interaction term between the planning method and the use of FWAs further indicates that this measure only has a significant influence if the fully digitized planning method is used. This means that the fully digitized planning method takes longer if no FWA is involved.

Eq 2 gives an overview of model formulae recommended for predictive use, including the model coefficients. Variables are defined in Table 2.

Time_F=Exp(4.67276+β1–(0.92686*log(STS))+β2–0.13509*log(Current_Pract)+β3+(0.86241*β4)) (2)

with

β1 = 0 for Cond = = easy;

β1 = 0.29267 for Cond = = moderate;

β1 = 0.57644 for Cond = = difficult;

β2 = 0 for PMS = = 3;

β2 = -0.905837 for PMS = = 5;

β3 = 0 for FWA = = with;

β3 = -0.14010 for FWA = = without;

β4 = 1 for PMS = = 5 AND FWA = = without;

β4 = 0 for everything else.

## 4. Discussion

Our study provides initial quantitative insights into the time requirements for skid trail planning. We analyzed natural, technical and personnel factors influencing the planning effort and developed an initial model to estimate the time requirement (and subsequently the costs) of skid trail planning. For evaluating the results and for informing potential future surveys, we critically reflect upon the following aspects of the study below: (i) the online survey and the process of selecting the participants, (ii) the degree of digitization of planning methods, (iii) the use of FWAs in transferring planning results to the field, (iv) the planner’s experience, and (v) the quality and the impact of the model.

### (i) The online survey and the selection process

Direct measurement of the time requirements of the planning process was considered impractical due to time and cost constraints. Therefore, a survey was chosen where participants estimated the time required for planning. To conduct the survey adequately and efficiently, it was carried out as an online survey.

It was challenging to find foresters skilled in planning and with sufficient experience to provide meaningful answers regarding time requirements. Therefore, we contacted appropriately trained and experienced forestry managers, asking them to fill out the questionnaire themselves and to pass it on to other similarly trained and experienced managers. In this regard, the two-stage selection underlying the survey was not random and not clearly determinable regarding the total target group. Thus, the sample size obtained was small despite the relatively high response rate of approximately 30%.

### (ii) Degree of digitization of planning methods

As planning methods are still in development, and to gain detailed insights into the practical approach, survey participants were asked to describe the processes they used rather than simply naming them. These descriptions of planning methods were stratified according to their degree of digitization, enabling the derivation of a corresponding variable with two categories: “partially digitized” and “fully digitized”. The degree of digitization was found to have a significant impact on the time requirement. With the fully digitized method, an average of more than 1 PSH_15_ ha^-1^ can be saved in skid trail planning. Thus, due to the labor-intensive nature of planning procedures, digitization offers significant cost-saving potential. These savings of about 1 PSH_15_ ha^-1^ amount to one to two million euro in total for a forestry office managing twenty thousand hectares (assuming 100% skid trail area and system costs of 50–100 euro PSH_15_^-1^).

### (iii) The use of FWAs in transferring planning results to the field

Most planning steps are primarily carried out by the forest managers alone; however, in approximately 36% of cases, a FWA is involved in transferring the planned route lines to the field (step 4 of planning). This leads to higher total personnel times (PPH_15_) or costs compared with solo work. In terms of system time (PSH_15_), we observed a time reduction–averaged over all terrain and stand conditions, as well as over all skid trail spacing situations–of 0.6 PSH_15_ ha^-1^ both on average and in terms of medians, or a savings of approximately 20%.

The regression analysis showed only in combination with the fully digitized planning method a significant benefit of using FWAs, which means that field assistance decreases the planning time of the team. It is surprising that planning time only increases without an FWA if fully digitized planning methods are used. One reason could be that the GPS signal is usually weak under canopy cover and requires some waiting time until a sufficient positional accuracy is given. The FWA then holds the receiver, and the experienced workforce can continue with the work during the waiting time. Another explanation is that if a single person must operate the receiver while concurrently completing other work steps, this has a negative influence on the productivity of these remaining work steps.

It is also possible that when work is done in teams, the primary focus is not just on reducing the time spent, but also on facilitating work and finding solutions during field work. Moreover, knowledge transfer to less experienced colleagues might be a further reason to make use of assistance. Furthermore, the available data set does not provide any information as to whether the work could not have been carried out without a FWA, or whether it would have taken even more time without one. Given that two-thirds of managers do not use FWAs, even in challenging conditions, it would be additionally necessary to examine whether they are essential in all cases or if there are potential areas for savings in this regard. This issue concerning field work assistance should be considered more thoroughly in the design of potential follow-up studies.

### (iv) Experience of the planner

The survey included questions on the planner’s expertise in terms of years of experience and scope in skid trail planning per year. The potential relevance of this factor emerged from feedback received from forest practitioners prior to the survey. As described in the results, the survey was completed by forest managers who, while adept in skid trail planning, had a wide range of experience (0.5–38 years). Since the statistical analysis did not show a significant influence of years of experience on the time requirement, a new variable was formed from the product of years of experience and the annual planning volume. However, no significant influence was detected with this variable either. We conclude that beyond a certain level of basic expertise in planning, additional years of experience do not markedly decrease the time requirement. However, an influence of the annual work volume was identified in our study. For the development of the practical model for estimating time requirements (see section 5: Conclusions), an individual time surcharge was included in case a planner lacked experience or knowledge in this domain.

### (v) Quality and impact of the model

The statistical analysis revealed large standard deviations in time requirements, which, in conjunction with the small sample size, result in low goodness-of-fit measures for the estimation model (adjusted r-squared: 0.30). The large variance can be explained first by the simple, user-friendly nature of this model, as a more complicated model indeed had an r-squared value of 0.51, and second by the use of a survey, in which the times had to be estimated. A third reason is that the conditions varied widely, and this was only partially covered by the classification into three categories (*Cond*). A fourth reason for the large variance is the lack of standardized methods. Everyone has developed their own method; even in training, no single best method is developed or taught. Therefore, we are convinced that a best practice could be established through the exchange of experiences and targeted workshops (or improved training), which in many cases would further increase the efficiency of planning.

To address the wide dispersion of the time requirements estimated by the survey participants, methods of ROB regression were applied (section 3.3: Regression models). As a result, the model was gradually improved. In this approach, all expected influencing factors are considered. Further influencing factors, such as the complexity of the planning task, were not examined here; however, they were not identified as missing predictors by the survey participants during the data collection process. The model enables a first estimation of the time requirement under various planning methods and operational conditions.

A validation of the model was planned but had to be abandoned due to insufficient participation/support from forestry practice (caused by capacity issues of respondents, especially due to calamity work).

Skid trail planning has a significant impact on timber production in practice: its quality strongly influences the performance and costs of timber harvesting, as well as the ecological effects of driving on the soil with logging machines. The time required for this planning and its costs can be estimated or calculated using our model. Its effort is important even under easy to moderate conditions (median 2.60–3.50 PSH_15_ ha^-1^) and considerable under difficult conditions (median 4.80 PSH_15_ ha^-1^). As an example of a rough cost estimate assuming system costs of 50–100 euro PSH_15_^-1^ and a harvesting volume of 500 m^3^ ha^-1^ in 50 years, a planning effort of 2.60–4.80 PSH_15_ ha^-1^ would correspond to a cost of 0.26–0.96 euro for each harvested solid cubic meter. This corresponds to roughly 1% of the harvesting costs per cubic meter. The unit costs, of course, depend heavily on capacity utilization, or the amount of wood produced.

## 5. Conclusions

Our study enables detailed insights into the planning process of skid trails in German and Swiss practice and its performance. Our model provides an estimation of the time needed for skid trail planning under specific forest conditions. To our knowledge, it is the first model of this type. Additionally, it indicates approaches for improving the planning process. Choices regarding the degree of digitization or the method and the deployment of personnel are starting points for reducing costs. According to our survey, planning requires an effort of approximately 3 to 4 PSH_15_ ha^-1^, with deviations possible depending on the specific situation in the stand. The relatively low costs of skid trail planning, i.e. less than one euro per solid cubic meter or about 1% of the harvesting costs, can open opportunities for considering quality aspects.

In the future, the question of the time required for skid trail planning in practice should be investigated on the basis of a larger amount of data. The use of auxiliary staff and the improvements of existing skid trail systems should also be examined more closely. The potential of the digitization of skid trail planning should be investigated in depth, regarding both planning effort and quality, to comprehensively assess the economic effects on timber harvesting and the ecological impact of the technical operations.

## Supporting information

S1 Questionnaire (DOCX)

S1 Table Overview of the analyzed response (= R) and predictor variables (= P). (XLSX)

S2 Table Model categories tested (PMS: simplified planning method variable, PMF: full planning method variable). (XLSX)

S3 Table Summary of the output from model 1a “PMSwo_Exp” (final model for predictive use). (XLSX)

S4 Table Time requirements of skid trail planning depending on personnel deployment, expressed in productive system hours (PSH_15_) and personnel hours (PPH_15_). (XLSX)

S1 Fig Residual plot for model 1a “PMSwo_Exp”. (TIFF)
