# Supplementary material for: A comprehensive analysis of time investment in skid trail planning for forest access
Source: PLoS One. 2025 Feb 4;20(2):e0317963. doi: 10.1371/journal.pone.0317963 (PMC11793781; doi:10.1371/journal.pone.0317963)
Supplement: S1 Questionnaire — (DOCX) [file pone.0317963.s001.docx]

*S1 Appendix: Questionnaire.*

**Survey on the Planning of Fine-scale Access with Skid Trails** (translated from German).

**Purpose of the Survey:** The aim is to create a model for estimating the time and cost required for planning fine-scale access with skid trails for the HeProMo/JuWaPfl model collection (available at wsl.ch and waldwissen.net).

**Definition:** Planning of fine-scale access with skid trails refers to both establishing new access routes and improving existing ones, integrating already present elements. This includes: (a) preparatory planning work in the office, and (b) field/stand checks to precisely determine and transfer the alignment of skid trails into the terrain. The practical establishment of skid trails through felling operations is generally not included, except when using directional lasers (see Question 2.3).

**Effort:** The estimated time to complete the questionnaire is approximately 20 minutes. We, Janine Schweier, Oliver Thees, and Marc Werder, sincerely thank you for taking the time and contributing your expertise to the practical forest research conducted by WSL.

This survey contains 20 questions.

1. **Personal Information/Experience**

How many years have you been working in your forest district/operation area, and have you been involved in the planning of skid trails?

Please enter your response here: • Years

1. **Planning Methods**

We would like to know which planning methods you normally employ. However, it is challenging to describe these methods or processes with a single term or a few words. This challenge can be mitigated by breaking down the entire process into individual steps and describing how these steps are executed. After describing the individual steps, we can clearly define your method. Please answer the questions regarding steps 2.1 and 2.5 and select the respective activity for steps 2.2–2.4.

*2.1 Preparatory Work in the Office*

We assume that the preparatory work in the office is carried out using: (a) remote-sensing-based maps (satellite images, orthophotos, LIDAR images) and (b) various topographic maps. If you use additional documents or follow a different approach, please document it here:

*2.2 Preparatory Work in the Field*

Checking the preparatory work in the office. Recording the existing skid trails using: Please select only one of the following answers:

• Hand mapping

• GPS recording

*2.3 Planning in the Office*

Planning of the entire skid trail system using:

Please select only one of the following answers:

• Paper map

• GIS

*2.4 Marking/Staking in the Field*

Verification, marking and recording of the planned skid trail system using:

Please select only one of the following answers:

• Staff, compass, paper map

• Staff, compass, mobile GPS

• Mobile GPS

• Directional laser

*2.5 Documentation in the Office*

We assume that documentation in the office includes (a) review and filing of recorded skid trail systems and (b) any additions and corrections to alignments (e.g. in the case of missing trail entries). If you perform additional tasks or follow a different approach, please document it here:

*Comment:*

Please enter your response here:

1. **Scope of Planning**

To what extent do you plan fine-scale access with skid trails each year?

Please enter your response here: • ha/year

What percentage of your planning involves:

• Planning new skid trails Percent

• Improvement of existing skid trails Percent

*Comment:*

Please enter your response here:

1. **Skid Trail Spacings**

What spacings (center to center) do you currently plan or have planned in the last five years?

Please select the appropriate spacings and estimate the corresponding percentage of the area planned for each spacing:

• 20m Percent

• 30m Percent

• 40m Percent

• Other: Percent

*Reason/Comment:*

Please enter your response here:

1. **Time Required for the Chosen Planning Process (refer to Question 2)**

Please estimate the time required (productive working time including interruptions of less than 15 minutes (PSH_15_)) for the four steps you selected under different conditions. We assume that the time estimates relate to the skid trail spacings you provided in question 4. If additional people are involved in the planning process, please provide their time estimates in the table below.

Definitions of conditions:

* Easy: e.g. flat terrain, leafless condition, clear, good visibility, no or minimal obstruction by vegetation and terrain features such as ditches and rocks

** Moderate: e.g. spring or autumn with little foliage, passages through young forest, restricted visibility

*** Difficult: e.g. pronounced slope, leafy condition, much young forest or many saplings, large proportion of conifers, significantly restricted visibility, high roughness of terrain, wet areas, consideration of many old skid trails

Your time estimates for planning in hours per hectare (h/ha):

|  | Easy  Conditions* | Moderate  Conditions** | Difficult  Conditions*** |
| --- | --- | --- | --- |
| Preparatory Work in the Office and Field (h/ha) |  |  |  |
| Planning in the  Office (h/ha) |  |  |  |
| Marking/Staking in the Field (h/ha) |  |  |  |
| Documentation in the  Office (h/ha) |  |  |  |

Time estimates of additional people involved in the planning process (e.g. foresters, forest technicians, assistants) in hours per hectare (h/ha):

|  | Number of Additional People Involved | Easy  Conditions* | Moderate  Conditions** | Difficult  Conditions*** |
| --- | --- | --- | --- | --- |
| Preparatory Work in the Office and Field (h/ha) |  |  |  |  |
| Planning in the  Office (h/ha) |  |  |  |  |
| Marking/Staking in the Field (h/ha) |  |  |  |  |
| Documentation in the  Office (h/ha) |  |  |  |  |

1. **Time Surcharge for Planning Effort**

In your opinion, what is the time surcharge compared with an experienced person (as a percentage) for the planning effort require by someone who:

1. is new to a forest district or area of operation and therefore has limited local knowledge?

Please enter your response here: • Percent

1. has little experience with the respective planning procedure?

Please enter your response here: • Percent

1. **Influence on the Planning Process**

Which of these factors has the greatest influence on the time required for the entire planning process?

Please rank the three factors (1 = greatest influence, 3 = least influence).

*Ranking*

• Experience (extent of skid trail plannings; local knowledge)

• Planning procedure (as per Question 2 selection)

• Conditions (visibility, terrain, vegetation, existing skid trails)

*Comment:*

Please enter your response here:

**Thank you for participating in this survey!**

__________________________________________________________________

S2 Table: Overview of the analyzed response (=R) and predictor variables (=P).

S3 Table: Model categories tested (PMS: simplified planning method variable, PMF: full planning method variable).

S4 Figure: Residual plot for Model 1a “PMSwo_Exp”.

*S5 Table: Summary of the output from Model 1a “PMSwo_Exp” (final model for predictive use).*
