# Supplementary figures and images for: A comprehensive analysis of time investment in skid trail planning for forest access
Source: PLoS One. 2025 Feb 4;20(2):e0317963. doi: 10.1371/journal.pone.0317963 (PMC11793781; doi:10.1371/journal.pone.0317963)

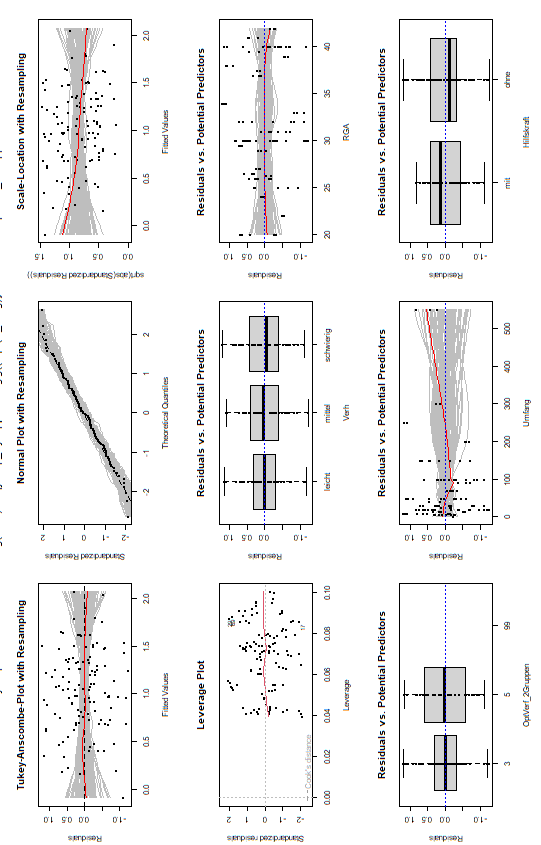

Supplement: S1 Fig — (TIFF) [file pone.0317963.s006.tiff]
